# The Waveform Fluctuation and the Clinical Factors of the Initial and Sustained Erythropoietic Response to Continuous Erythropoietin Receptor Activator in Hemodialysis Patients

**DOI:** 10.1100/2012/157437

**Published:** 2012-04-19

**Authors:** Wen-Sheng Liu, Yueh-Lin Wu, Szu-Yuan Li, Wu-Chang Yang, Tzen-Wen Chen, Chih-Ching Lin

**Affiliations:** ^1^School of Medicine, National Yang-Ming University, Taipei 112, Taiwan; ^2^Division of Nephrology, Department of Medicine, Taipei Veterans General Hospital, Taipei 112, Taiwan; ^3^Division of Nephrology, Department of Medicine, Taipei City Hospital, Zhong-Xing Branch, Taipei 103, Taiwan; ^4^Division of Nephrology, Department of Medicine, Taipei City Hospital, Zhong-Xiao Branch, Taipei 115, Taiwan; ^5^Division of Nephrology, Department of Medicine, Taipei Medical Univeristy Hospital, Taipei 110, Taiwan; ^6^Department of Medicine, School of Medicine, College of Medicine, Taipei Medical Univeristy, Taipei 110, Taiwan

## Abstract

*Objectives*. Erythropoiesis-stimulating agents (ESA) are the main treatment for anemia in hemodialysis (HD) patients. We evaluated factors determining the response after treatment of a new ESA (continuous erythropoietin erythropoietin receptor activator (CERA)). *Methods*. 61 HD patients were classified by their response at two different timings. First, patients whose hematocrit (Hct) increased 1.5% in the first week were defined as initial responders (IR, *n* = 16). We compared several parameters between IR and the rest of the study subjects (non-IR, *n* = 45). Second, patients whose Hct increased 2% in the 4th week were defined as sustained responders (SR, *n* = 12), and we did a similar comparison. *Results*. The Hct showed a waveform fluctuation. Compared with the rest, IR had significantly lower platelet counts and higher levels of ferritin, total protein, total bilirubin, and serum sodium, while SR had significantly lower levels of C-reactive protein and low-density lipoprotein (All *P* < 0.05). In comparison with the rest, higher Hct persisted for 10 weeks in SR but only for two separate weeks (the 1st and 7th week) in IR. *Conclusions*. The initial and sustained erythropoietic responses are independent from each other and are associated with different factors. Treatment focusing on these factors may improve the response.

## 1. Background

Anemia is a frequently encountered problem in chronic kidney disease (CKD). Erythropoiesis-stimulating agents (ESA) are the mainstay treatment [1]. The features of therapy are (1) erythroid response is dose dependent but variable at a given dose and (2) infections and inflammation may blunt the response to ESA [1]. Iron deficiency is the most frequently encountered cause of suboptimal response [2]. Improving iron status allows for lower ESA doses [3]. Other possible factors in ESA responsiveness include dialysis adequacy and usage of medications such as HMG-CoA reductase inhibitors (statins), angiotensin-converting enzyme inhibitors (ACEI), and angiotensin II receptor blockers (ARB) [4]. Management of anemia in chronic renal insufficiency (CRI) cases represents an important medico-economic challenge because of the large number of patients and the cost of ESA. In order to minimize the inconvenience and discomfort, ESA with a longer dose interval is indicated [5]. In 2007, CERA (continuous erythropoietin receptor activator) (Mircera, Roche) was approved. CERA is effective for renal anemia. CERA has a considerably longer half-life, making it the only ESA licensed for once-a-month dosing [6]. In 2009, CERA became available in Taiwan. However, to date, no study has been performed on the factors influencing the efficacy of this new ESA in Taiwan. We aimed to assess the influencing factors [7].

## 2. Methods

### 2.1. Study Patients

This was a prospective observational study approved by the institutional review board. Seventy hemodialysis patients at Taipei Veterans General Hospital receiving CERA as their treatment of ESA after April 1, 2010 were enrolled in this study. Before enrollment into this study, all the study patients had received Aranesp (DPO) as the only choice of their ESA treatment, thus a wash-out period for at least 10 days are necessary prior to the first dose of CERA. The half-life of DPO is 21 h [8]. Thus, 10 days is sufficient to wash out its previous effect (5 × 21 h = 105 h = 4.37 days). Nine patients were excluded due to having active bleeding (major trauma, gastric ulcer bleeding, or surgery), blood transfusion, or additional ESA injection within follow-up period after CERA use.

Demographic and clinical data such as age, gender, body weight, duration of dialysis, diabetes and hepatitis status, adequacy index of HD (Kt/V, determined by Daugirdas method), artificial kidney (AK) clotting, and medication (iron supplements, ACEIs, ARBs, and statins) were obtained from the medical records. Laboratory parameters are gathered at the beginning of the month prior to CERA use, which included hemogram: white blood cell (WBC) count, red blood cell (RBC) count, hematocrit (Hct), and platelet count. Iron storage status: serum iron, total iron-binding capacity (TIBC), and transferrin saturation (TSAT), ferritin. Biochemical profile: protein, albumin, total cholesterol, triglyceride, uric acid, high-density lipoprotein (HDL), low-density lipoprotein (LDL), glucose, blood urea nitrogen (BUN), creatinine (Cr), sodium (Na), potassium (K), chloride (Cl), calcium (Ca), phosphate (P), serum intact parathyroid hormone (iPTH), total bilirubin, alkaline phosphatase (ALK-P), *γ*-glutamyl transferase (GGT), alanine transaminase (ALT), and aspartate transaminase (AST). Inflammation marker: C-reactive protein (CRP).

All patients were followed up to December 31, 2010, or until they discontinued CERA as their ESA. In Taiwan, all ESRD patients receive CERA 100 micrograms (*μ*g) in the beginning of each month under the guideline set by Taiwan national health insurance system. The average body weight in 4 groups (IR, non-IR, SR, and non-SR) are all similar and in normal distribution (Table 1). So, their Hct can be a good reflection of their response to CERA.

### 2.2. Definition of Initial Response and Sustained Response

**Figure 1:**
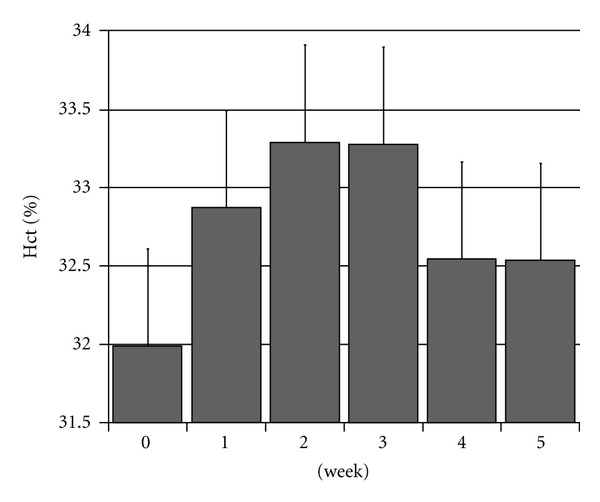
The changes of hematocrit during the first 5 weeks after CERA use. The changes of hematocrit in 61 hemodialysis patients during the first 5 weeks. (The mean value and standard deviation of Hct of HD patients are displayed weekly.)

The Hct under CERA treatment showed a waveform response, which showed a significant increase at the first week and then had a significant fall at the fourth week (Figure 1). So, we classified their erythropoietic response after treatment of CERA according to the following criteria at two different timings. First, patients whose hematocrit (Hct) increased by 1.5% in the first week were defined as initial responders (IR) (*n* = 16). We compared IR and the rest of the study group (*n* = 45) in the factors listed above. Second, patients whose Hct increased by 2% in the 4th week were defined as sustained responders (SR) (*n* = 12), and we did the similar comparison.

### 2.3. Statistical Analysis

Continuous data were expressed as mean ± standard deviation. If the continuous variable was not in normal distribution, we expressed it in interquartile range. Statistical analysis was performed using SPSS 18.0 for Windows web version. The *χ* ^2^ test was used for categorical variables and the *t* test for continuous variables. A *P* value less than 0.05 was regarded as statistically significant.

## 3. Results

### 3.1. Definition of Initial Response and Sustained Response

70 patients enrolled, and with excluding nine who met the exclusion criteria, 61 were for analysis. Their mean Hct showed a significant rise in the first week and reach a plateau during the 2nd-3rd week, then had a significant fall on the 4th week (Figure 1). Our autoanalyzer is Beckman Coulter LH 750 with a coefficient of variance of 2%. So, the standard derivation of Hct measurement is (31%  ∗ 0.02) 0.62%. The mean Hct increase at the first week is 0.88% and 1.30% at week 2 and 3. So, we defined the good initial response is 1.50% (mean change + SD = 0.88% + 0.62%) on the 1st week, and the good sustained response is maintained a 2% (maximal change + SD = 1.30% + 0.62%) increase in Hct on the 4th week. In 61 patients, there are 16 initial responders (IR) and 45 who are not initial responders (non-IR). And there are 12 sustained responders (SR) and 49 who are not sustained responders (non-SR). Then we compare the difference in the factors listed in method between IR and non-IR and also SR and non-SR.

### 3.2. Comparisons between Initial Responders (IR) and Non-IR, Sustained Responder (SR) and Non-SR

The demographic and clinical parameters are of no significant difference between IR and non-IR, neither between SR and non-SR (Table 1). However, IRs had significantly lower platelet counts (*P* = 0.046) and higher ferritin (*P* = 0.046), TP (*P* = 0.026), Na (*P* = 0.024), and total bilirubin levels (*P* = 0.039), with borderline significantly higher albumin levels (*P* = 0.060) than the corresponding values in the rest of the study group, non-IR (*n* = 45). Sustained responders (SR) (*n* = 12) had a significantly lower levels of LDL (*P* = 0.043) and lower CRP level (*P* = 0.049) with borderline significantly lower WBC count (*P* = 0.077) than the corresponding values in the rest of the study group, non-SR (*n* = 49). All other parameters were of no significant difference (Table 2). 

The Chi-square test concerning the association of IR and SR revealed a *P* value of 0.715, which showed no significant association. Further analysis of multivariate linear regression between Hct change at week 1 and week 4 and their significant influencing factors mentioned above both showed good correlation (Table 3). This further confirmed that there are different factors influencing the initial and sustained responses of CERA.

**Figure 2:**
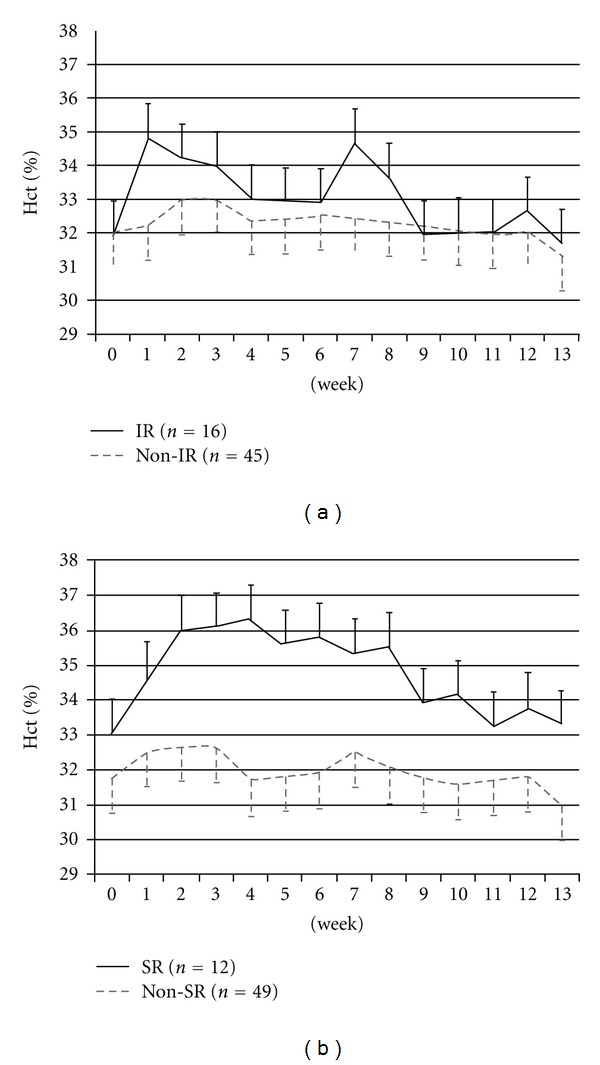
The Variation of hematocrit between IR and non-IR (a) and SR and non-SR (b). (a) Comparison of changes of Hematocrit after treatment of CERA between initial responders (IR) and the rest of the study group (non-IR), (b) similar comparison between sustained responders (SR) and the rest of the study group (non-SR). (The mean value and standard deviation of Hct of HD patients are displayed weekly.)

We followed up the patients Hct levels weekly up to 13 weeks. Compared to the rest of the study group, the IRs had significantly higher Hct only at the 1st and 7th week (which is about 2 weeks after the second CERA dose was given). However, the SRs had significantly elevated Hct (*P* < 0.05) from the 1st to 10th week after CERA use (Figure 2). There was no significant difference after the 11th week. The pattern of erythropoietic response was similar in the first two months (1st to 10th week). 

## 4. Discussion

We found that the weekly Hct showed a waveform response under CERA treatment and was different week by week. So, it would be misleading if we only choose certain time point of Hct to define the response of CERA without a timely manner view. The Hct showed a significant rise at the first week, reached plateau at the second and the third week, and had a significant fall on the fourth week. We can separate initial responders (IR) and sustained responders (SR) from the rest of study patients by their Hct at two different time points. This could not be conducted by other ESA with shorter dose interval, because we cannot access patients' Hct at four different timings in one dose interval.

This study discloses the clinical factors predicting the initial and sustained erythropoietic response after receiving treatment of CERA in HD patients. Compared with the rest, IR had significantly lower platelet counts and higher levels of ferritin, total protein, total bilirubin, and serum sodium with borderline higher albumin level, while SR had significantly lower levels of CRP and LDL with borderline lower WBC counts. These parameters collected before CERA treatment can predict the ESA response over the following 2 months.

As for the determining factors of the initial response, initial responders (IR) had low platelet counts. This may represent more erythroblasts in bone marrow. Expanded erythropoiesis appears to exert a negative impact upon platelet production in an animal study [9]. It is consistent with stem-cell competition between erythroid and megakaryocytic development. IR also had higher iron storage [10]. The higher the ferritin level was, the higher the initial response would be. However, it was not related to the sustained response. Studies also showed that ferritin levels are poor predictors of positive response [10, 11] and our study may explain the reason, because ferritin is also a marker of inflammation [12]. Total protein and albumin, markers for nutrition status, also had significant correlation with the initial ESA response [13, 14]. Albumin level also increases in dehydration, which correlated with higher Hct. The higher level of Na may also reflect dehydration within the patients' body [15] and hence a higher Hct. Total bilirubin was significant higher in the IR group but not other liver function profiles. So, it is unlikely from a hepatic origin. According to the study by Lin et al., the antiinflammatory effect of far infrared therapy which inhibits vascular inflammation through the induction of HO-1 actually comes from bilirubin-related inhibition of cytokine-mediated expression of adhesion molecules in endothelial cells [16]. Higher total bilirubin implies a higher anti-inflammatory and anti-oxidant effect. In addition, ESA treatment may produce oxidative stress in association with elevation in BP [17]; therefore, patients with higher total bilirubin can benefit more from ESA treatment.

For sustained responder (SR), their lower WBC count and CRP level may reflect lower inflammation status [1]. In addition, LDL was also lower in the SR and this finding could be explained as follows. First, according to the report by Shalev et al., cholesterol would be consumed during erythropoiesis [18], hence lower cholesterol level may be associated with better erythropoietic response. They found all patients with chronic anemia and increased erythropoietic activity had hypocholesterolemia, so they suggest that the high-erythropoietic activity-associated hypocholesterolemia is due to increased cholesterol requirements by the proliferating erythroid cells. Second, statins, cholesterol-lowering agents, had been reported to improve ESA response [4]. According to the study by Chiang et al., proinflammatory cytokines included interleukin-6 and tumor necrotic factor-alpha levels decreased after statin therapy, and thus may improve ESA hyporesponsiveness in dialysis patients. Third, LDL was demonstrated to have good correlation with CRP, thus it could be taken as a marker of inflammation [19] and would be associated with a negative impact on the survival of patients with CKD [20].

As for factors related to treatment and medication, all factors were similar in IR and non-IR, also in SR and non-SR. Although higher dialysis clearance, iron supplements, statins had been reported to improve ESA response [4] and ACEI/ARB may have a negative impact [21], we did not find any significant difference among these aspects. This may due to the following facts: quality controls for dialysis in Taiwan ensure that all dialysis patients meet the standard KT/V. Statins are given to the high-risk HD patients following the guidelines of national health insurance in Taiwan. In our hospital, iron supplements are given only to patients with ferritin level <200 ng/mL. ACEI/ARBs are given according to guidelines in Joint National Committee −7 with individual adjustment [22]. Thus, it is not the treatment (such as statins or iron supplement) but the parameters of the clinical response (LDL and ferritin) to the above-mentioned treatment that would influence the erythropoietic response.

Previous study by Locatelli et al. showed similar results that higher albumin and iron status were associated with better ESA response and lower ESA requirement, while higher CRP was associated with higher ESA requirement [23]. But they did not differentiate the influencing factors associated with the responses of Hct at different timings.

The strength of our study, from the viewpoint of practicability, is that the factors we used for predicting the erythropoietic effect of CERA are all routine biochemical parameters. The assessment is easy, and the criteria are simple. It provides a simple and practical way to predict the response of CERA up to 10 weeks later.

There are some limitations for this study. First, this is an open-label, observational study, so patients are aware of the change of ESA from DPO to CERA in this study. However, it is not necessary to conduct this study in a blind way, because all study patients received CERA as the choice of their ESA treatment. Second, the cohort and the follow-up period may not be big or long enough to detect minor factors. However, to our knowledge, it is by far the largest population study concerning CERA in HD patients in Taiwan.

## 5. Conclusions

This is the first study concerning the clinical factors predicting the initial and sustained erythropoietic response after receiving treatment of CERA in HD patients. With long half-life of CERA, we can separate the initial and sustained response on a weekly scale. The initial and sustained erythropoietic responses after treatment of CERA are independent from each other and they are, respectively, associated with different factors, which also showed good predicting power of ESA response over two months. The sustained response is related to inflammatory status and is more important than the initial response or serum ferritin. More studies are needed to investigate whether multidisciplinary treatment focusing on correcting the above-mentioned risk factors may improve the erythropoietic response to CERA in HD patients in the future.

## Figures and Tables

**Table 1:** Comparison of the baseline demographic and clinical parameters among all study patients, initial responders (IR), the rest of the study patients (Non-IR), sustained responders (SR), and the rest of the study patients (Non-SR) after treatment of CERA.

Case number	All (*n* = 61)	IR (*n* = 16)	Non-IR (*n* = 45)	SR (*n* = 12)	Non-SR (*n* = 49)
Age (years)	69.05 ± 15.28	67.50 ± 17.31	69.60 ± 14.66	70.58 ± 10.82	68.67 ± 16.26
Male *n* (%)	28 (46%)	8 (50%)	20 (44%)	8 (67%)	20 (41%)
HD duration (months)	81 (44.5–118.25)	82 (38–116)	81 (45–121.5)	83.5 (50.25–118.75)	81 (41.75–118.25)
BW (kg)	57.26 ± 11.19	58.35 ± 13.46	57.39 ± 10.51	55.55 ± 12.05	58.18 ± 11.02
DM *n* (%)	28 (46%)	7 (44%)	21 (47%)	8 (67%)	20 (41%)
Hepatitis *n* (%)	4 (7%)	0 (0%)	4 (9%)	1 (8%)	3 (6%)
Kt/V	1.64 ± 0.23	1.57 ± 0.28	1.66 ± 0.21	1.62 ± 0.22	1.64 ± 0.24
Clotting during HD *n* (%)	9 (15%)	1 (7%)	8 (17%)	1 (8%)	8 (16%)
Iron supplement *n* (%)	5 (8%)	2 (13%)	3 (7%)	1 (8%)	3 (6%)
ACEI/ARB *n* (%)	30 (49%)	9 (56%)	21 (47%)	5 (42%)	25 (51%)
Statins *n* (%)	18 (30%)	6 (38%)	12 (27%)	5 (42%)	13 (27%)

HD, hemodialysis; BW, body weight; ACEI/ARB, angiotensin-converting enzyme inhibitors/blockers of angiotensin II receptor, type I.

**Table 2:** Comparison of the baseline laboratory parameters among all study patients, initial responders (IR), the rest of the study patients (Non IR), sustained responders (SR) and the rest of the study patients (Non-SR) after treatment of CERA.

Case number	All (*n* = 61)	IR (*n* = 16)	Non-IR (*n* = 45)	SR (*n* = 12)	Non-SR (*n* = 49)
WBC count (10^3^/mm^3^)	6200 (5250–7300)	5750 (4925–6675)	6500 (5250–7350)	5750 (4850–6100)	6600 (5300–7350)
RBC count (10^6^/mm^3^)	3.39 ± 0.38	3.35 ± 0.37	3.41 ± 0.38	3.34 ± 0.22	3.41 ± 0.40
Hct (%)	31.78 ± 4.15	31.99 ± 3.73	31.71 ± 4.33	32.70 ± 2.27	31.56 ± 4.48
Platelet count (10^3^/*μ*L)*	179.21 ± 50.64	162.31 ± 34.93	185.22 ± 54.23	180.00 ± 55.10	179.02 ± 50.10
Iron (*μ*g/dL)	57.39 ± 25.95	67.81 ± 36.50	53.69 ± 20.27	50.25 ± 14.31	59.14 ± 27.91
TIBC (*μ*g/dL)	209.33 ± 36.83	213.69 ± 39.56	207.78 ± 36.16	207.67 ± 31.61	209.73 ± 38.30
TSAT (%)	28 ± 14	32 ± 20	26 ± 11	24± 6	29 ± 15
Ferritin (ng/mL)*	371 (224.5–578.5)	475 (315.5–820.5)	361 (204.5–543.5)	353 (240.75–460)	375 (220–596)
Total protein (g/dL)*	6.82 ± 0.54	7.03 ± 0.56	6.74 ± 0.52	6.90 ± 0.60	6.80 ± 0.53
Albumin (g/dL)	3.93 ± 0.38	4.08 ± 0.29	3.87 ± 0.40	3.93 ± 0.34	3.93 ± 0.39
Total cholesterol (mg/dL)	184.88 ± 49.90	199.14 ± 50.16	174.90 ± 49.81	156.25 ± 59.83	193.69 ± 45.48
Triglyceride (mg/dL)	119.5 (75.00–213.75)	133 (81.00–230.00)	103 (70.50–200.00)	130.5 (78.75–213.75)	119.5 (71.25–213.50)
Uric acid (mg/dL)	8.17 ± 1.10	8.30 ± 1.17	8.08 ± 1.11	7.33 ± 1.27	8.43 ± 0.95
HDL (mg/dL)	45.93 ± 19.99	46.31 ± 15.49	45.79 ± 21.62	43.27 ± 12.40	46.55 ± 21.43
LDL (mg/dL)^+^	101.34 ± 42.399	98.13 ± 44.17	102.57 ± 42.19	79.45 ± 23.45	106.47 ± 44.35
Glucose (mg/dL)	156.18 ± 89.89	135.44 ± 76.78	163.56 ± 93.79	130.00 ± 92.84	162.59 ± 88.95
BUN (mg/dL)	75.79 ± 21.54	80.63 ± 27.13	74.07 ± 19.24	75.25 ± 25.26	75.92 ± 20.82
Cr (mg/dL)	11.14 ± 2.38	11.52 ± 1.96	11.01 ± 2.52	10.97 ± 2.42	11.19 ± 2.39
Na (mmol/L)*	136.43 ± 3.81	138.25 ± 3.91	135.78 ± 3.59	136.92 ± 3.40	136.31 ± 3.92
K (mmol/L)	4.7 ± 0.61	4.72 ± 0.60	4.70 ± 0.625	4.68 ± 0.55	4.71 ± 0.63
Cl (mmol/L)	95.84 ± 4.07	97.06 ± 4.57	95.40 ± 3.84	95.92 ± 2.27	95.82 ± 4.42
Ca (mg/dL)	9.49 ± 0.91	9.58 ± 0.85	9.46 ± 0.94	9.51 ± 0.74	9.49 ± 0.96
P (mg/dL)	4.62 ± 1.52	4.64 ± 1.53	4.62 ± 1.53	4.18 ± 1.18	4.73 ± 1.58
iPTH (pg/mL)	109.00 (50.08–277.00)	180.50 (94.27–381.75)	91.43 (41.13–179.00)	85.16 (28.92–269.00)	121.50 (53.48–293.50)
T.bili (mg/dL)*	0.23 ± 0.11	0.28 ± 0.139	0.21 ±.099	0.24 ± 0.16	0.23 ± 0.10
ALK-P (U/L)	82.59 ± 28.39	82.43 ± 25.86	82.70 ± 31.41	97.25 ± 21.08	78.08 ± 29.49
GGT (U/L)	17.00 (17.00–27.00)	18.50 (12.25–26.50)	17.00 (13.00–28.50)	21.50 (16.25–31.25)	17.00 (12.00–27.00)
ALT (U/L)	16.92 ± 8.19	17.38 ± 7.89	16.76 ± 8.38	20.92 ± 14.11	15.94 ± 5.77
AST (U/L)	16.82 ± 8.09	16.06 ± 6.59	17.09 ± 8.61	19.67 ± 12.76	16.12 ± 6.48
CRP (mg/dL)^+^	1.41 (0.31–3.90)	2.23 (0.31–7.63)	0.90 (0.30–2.34)	0.90 (0.18–2.58)	1.57 (0.31–4.19)

(*P < 0.05 for the comparison between IR and non-IR; ^+^ *P* < 0.05 between SR and non-SR).

WBC, white blood cell; RBC, red blood cell; Hct, hematocrit; TIBC, total iron-binding capacity; TSAT, transferrin saturation; HDL, high-density lipoprotein; LDL, low-density lipoprotein; BUN, blood urea nitrogen; Cr, creatinine; Na, sodium; K, potassium; Cl, chloride; Ca, calcium; P, phosphate; iPTH, serum intact parathyroid hormone; T. bili, total bilirubin; ALK-P, alkaline phosphatase; GGT, *γ*-glutamyl transferase; ALT, alanine transaminase; AST, aspartate transaminase; CRP, C-reactive protein).

**Table 3:** The multivariate linear regression model of factors associated with Hct change in HD patients after treatment of CERA in the 1st and 4th week respectively.

Hct change in the 1st week	B estimate	*P* value	*r* ^2^ = 0.408
Ferritin (for each 1 ng/mL increase)*	0.002	0.0002	
Na (for each 1 mEq/L increase)*	0.170	0.003	
Total protein (for each 1 g/dL increase)*	1.084	0.009	
Total bilirubin (for each 1 mg/dL increase)*	2.564	0.012	
Platelet (for each 1 10^3^/*μ*L increase)	−0.0047	0.292	

Hct change in the 4th week	B estimate	*P* value	*r* ^2^ = 0.215

LDL (for each 1 mg/dL increase)*	−0.041	0.0004	
CRP > 4 mg/dL or not*	−0.193	0.047	

*For *P* < 0.05.

B, unstandardized regression beta coefficient; Na = sodium; LDL, low density lipoprotein; CRP, C-reactive protein.
